# High spatiotemporal resolution data from a custom magnetic tweezers instrument

**DOI:** 10.1016/j.dib.2020.105397

**Published:** 2020-03-12

**Authors:** Eugeniu Ostrofet, Flávia S. Papini, David Dulin

**Affiliations:** Junior Research Group 2, Interdisciplinary Center for Clinical Research, Friedrich-Alexander-University Erlangen-Nürnberg (FAU), Cauerstraße 3, 91058 Erlangen, Germany

**Keywords:** Single molecule biophysics, Force spectroscopy, Magnetic tweezers, High spatiotemporal resolution

## Abstract

Gene expression is achieved by enzymes as RNA polymerases that translocate along nucleic acids with steps as small as a single base pair, i.e., 0.34 nm for DNA. Deciphering the complex biochemical pathway that describes the activity of such enzymes requires an exquisite spatiotemporal resolution. Magnetic tweezers are a powerful single molecule force spectroscopy technique that uses a camera-based detection to enable the simultaneous observation of hundreds of nucleic acid tethered magnetic beads at a constant force with subnanometer resolution [1,2]. High spatiotemporal resolution magnetic tweezers have recently been reported [3–5]. We present data acquired using a bespoke magnetic tweezers instrument that is able to perform either in high throughput or at high resolution. The data reports on the best achievable resolution for surface-attached polystyrene beads and DNA tethered magnetic beads, and highlights the influence of mechanical stability for such assay. We also present data where we are able to detect 0.3 nm steps along the z-axis using DNA tethered magnetic beads. Because the data presented here are in agreement with the best resolution obtained with magnetic tweezers, they provide a useful benchmark comparison for setup adjustment and optimization.

Specifications table

**Table:** 

Subject	Biophysics
*Specific subject area*	Single-molecule force spectroscopy using magnetic tweezers
*Type of data*	Figure, image, graph, table
*How data were acquired*	Custom built magnetic tweezers setup interfaced using a custom software written in Labview 2016 for three-dimensional bead position tracking.
*Data format*	Analyzed, raw
*Parameters for data collection*	Three types of measurement were performed: (1) tracking the 3D position of surface-attached 3 µm diameter polystyrene beads (reference beads) (2) tracking the 3D position of a magnetic bead (M-270 Streptavidin-coated, 2.8 µm diameter, Thermofisher) tethered by a 300 bp double-stranded DNA under constant force, (3) tracking the position of either a reference bead or a tethered magnetic bead while performing sub-nanometer steps using a piezo stage. Magnification of the objective, acquisition speed and shutter time were modified as described in (Supplementary Table 1).
*Description of data collection*	The bead position was acquired using a MT setup interfaced using custom Labview 2016 routines. Frame numbers, time stamp and x,y,z positions of the beads were saved as a tab delimited .txt file (raw data). The x, y, z positions of the beads were not drift corrected. When force was applied during the measurement, two more .txt files were generated: one describing the magnet position and the residence time at each position and a second containing a time stamp of magnet movement. Data were analyzed and plotted using Python.
*Data source location*	Interdisciplinary Center for Clinical Research, Friedrich Alexander University Erlangen-Nürnberg (FAU) IZNF, Cauerstrasse 3, 91,058, Erlangen, Germany Latitude 49.5719, longitude 11.0272. GPS: N 49 34.316, E 11 01.635
*Data accessibility*	Direct URL to data: https://data.mendeley.com/datasets/r5sk6z3v9k/draft?a=36f3f0d5–1545–45b2-b1e7-7486ebaf7fd2

## Value of the data

• The data reported here provide a benchmark of the best achievable spatiotemporal resolution of magnetic tweezers for a surface-attached reference bead and a DNA tethered magnetic bead.
• The data will be useful to evaluate the resolution of magnetic tweezers, and other camera-based detection assays, such as acoustic force spectroscopy and tethered particle motion.
• The data identifies several sources of noise that deteriorate the spatiotemporal resolution of magnetic tweezers.

## 1 Data

**Fig. 1:**
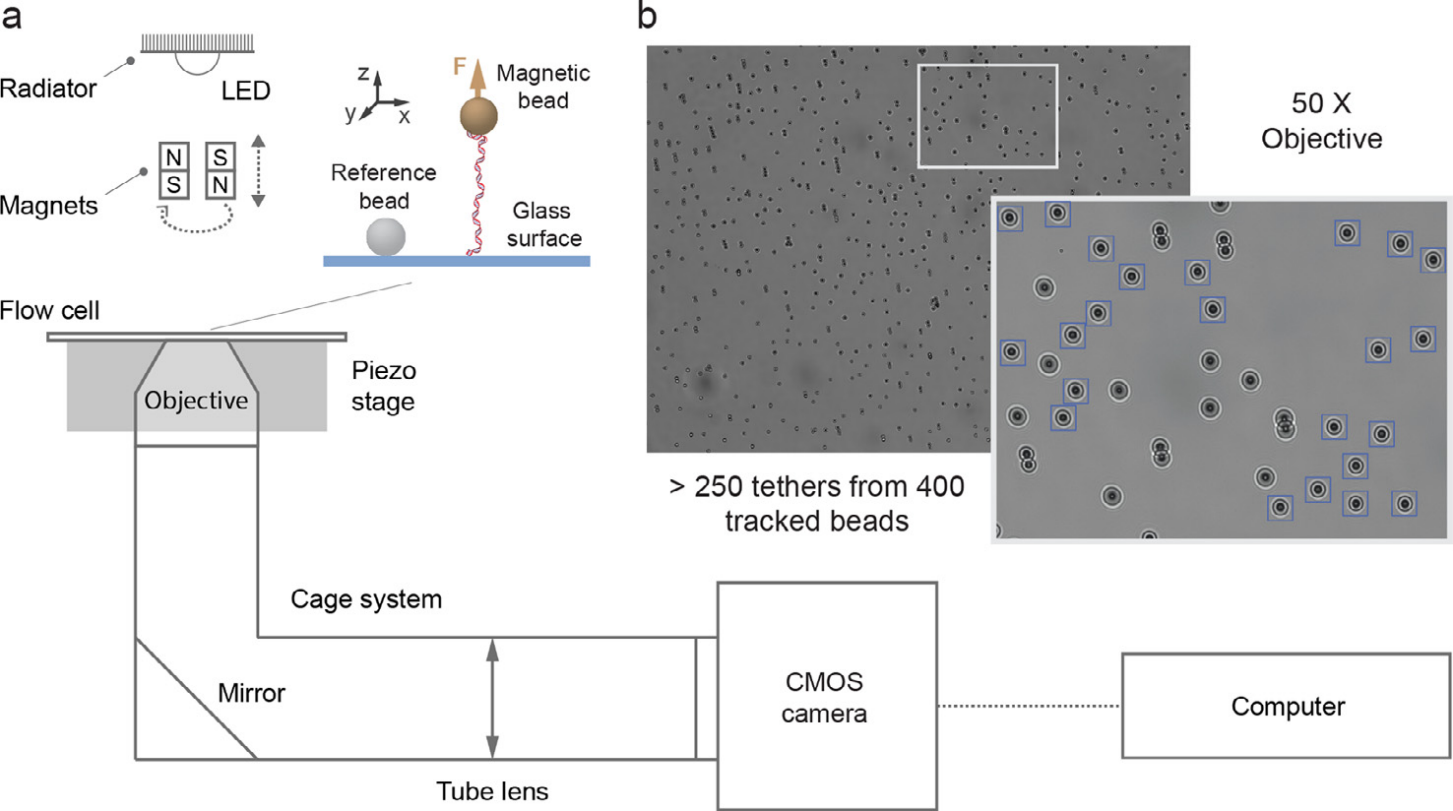
Magnetic tweezers enable high throughput single molecule data acquisition. (a) Schematic of the magnetic tweezers instrument. The DNA molecule is labeled at one end with a biotin and at the other end with two digoxigenins to tether a streptavidin-coated magnetic bead to the anti-digoxigenin coated glass coverslip surface of the flow cell. The mechanical drift is corrected by subtracting to the magnetic bead the position of a surface-attached reference bead. (b) Typical field of view for short DNA constructs (< 1 µm) tethered by 2.8 µm diameter magnetic beads in 50x magnification. The blue squares in the zoom of the image highlight the selected DNA tethered magnetic beads. (For interpretation of the references to colour in this figure legend, the reader is referred to the web version of this article.)

**Fig. 2:**
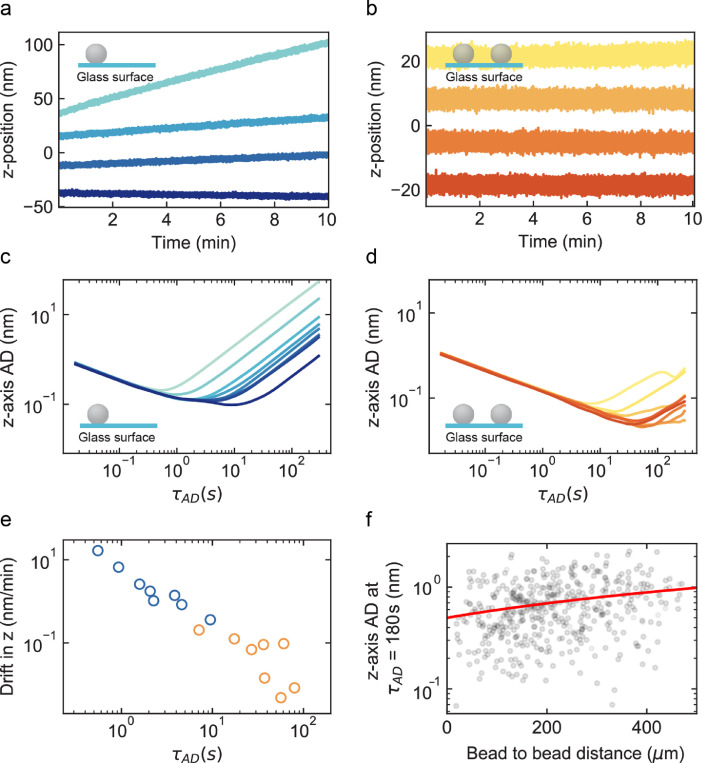
The initial mechanical drift limits the absolute resolution of the assay. (a) Every second trace out of eight successive recordings of the z-axis position of one 3 µm surface-melted polystyrene reference bead with mechanical drift varying from 0.36 nm/min (dark blue) to 16.65 nm/min (light blue). (b) Same bead and conditions as in (a) after mechanical drift correction by subtracting the z-axis position of another reference bead (light orange corresponds to light blue and dark orange to dark blue from panel (a). In (a) and (b), an offset was computationally added to separate and distinguish the traces. (c) Allan deviation (AD) of the position along the z-axis of the eight successive recordings of the surface-melted 3 µm polystyrene bead of (a) and (b). Same color code as in (a). (d) Same as (c) after subtraction of the position along the z-axis of another reference bead (same color code as in (b)). (e) *τ_AD_* for which the Allan deviation is minimal as a function of the amplitude of the mechanical drift in case of subtracted (orange) and non-subtracted (blue) reference bead. (f) Drift corrected AD along the z-axis at $\tau_{AD}=180\;s$ for different pairs of reference bead in the field of view with different bead-to-bead distance (*N*=30 reference beads, i.e., 841 different pairs). The red line is a linear fit with a slope of 0.0009 nm/µm. (For interpretation of the references to colour in this figure legend, the reader is referred to the web version of this article.)

The data are recorded using a versatile magnetic tweezers instrument that can be used either in high throughput or high-resolution mode [2] (Fig. 1(a)). The imaging path is fully tubed to prevent image distortion from airflow (Fig. 1(a)), and a box further insulates the instrument, while keeping the camera (the main heat source) isolated from the microscope stage. The high-throughput mode enables the simultaneous observation of ∼400 beads at 58 Hz (Fig. 1(b)), of which more than half passes the tether selection criteria, i.e., response to force increase and bead rotation (inset, Fig. 1(b)). The bead position is extracted in real-time using a Labview custom routine [1]. We have recorded the z-axis position of a surface-attached reference bead in eight successive experiments and we represent in Fig. 2(a) and (b) every second traces, without and with subtracting the z-axis position of another surface-attached bead, respectively. To quantify the mechanical drift as a function of the duration of the acquisition, we estimate the fully overlapping Allan deviation (AD) of the traces from Fig. 2(a) and (b) (Fig. 2(c) and (d), respectively). The Allan deviation is one-half the average difference in position between adjacent intervals of length *τ_AD_* over all intervals of length *τ_AD_* [3,6]. The AD decreases as 1/√*τ_AD_* until it increases at longer *τ_AD_*, indicating that the drift is now dominating the noise at longer integration time. Therefore, the AD of traces from experiment presenting a large drift (Fig. 2(a)) demonstrates a minimum at shorter *τ* than the ones that drift less, e.g., light blue vs. dark blue, respectively, in Fig. 2(c). Reference bead position subtraction does not completely correct for the mechanical drift observed in Fig. 2(b), as the experiments presenting the largest drift in Fig. 2(a) and (c) still demonstrate the largest drift after correction in Fig. 2(b) and (d). This behavior is further underlined by the *τ_AD_* value at which AD of a given trace in a given experiment is minimum: though *τ_AD_* diminishes with reference subtraction, the dispersion remains large, indicating that the initial drift still affects the resolution (Fig. 2(e)). We interrogate whether the distance between the two beads we used to perform the measurements as in Fig. 2(b) explains the residual drift we observe in Fig. 2(d) and (e). We observe a small correlation between the AD at $\tau_{AD}=180\;s$ and the bead-to-bead distance (Fig. 2(f)).

**Fig. 3:**
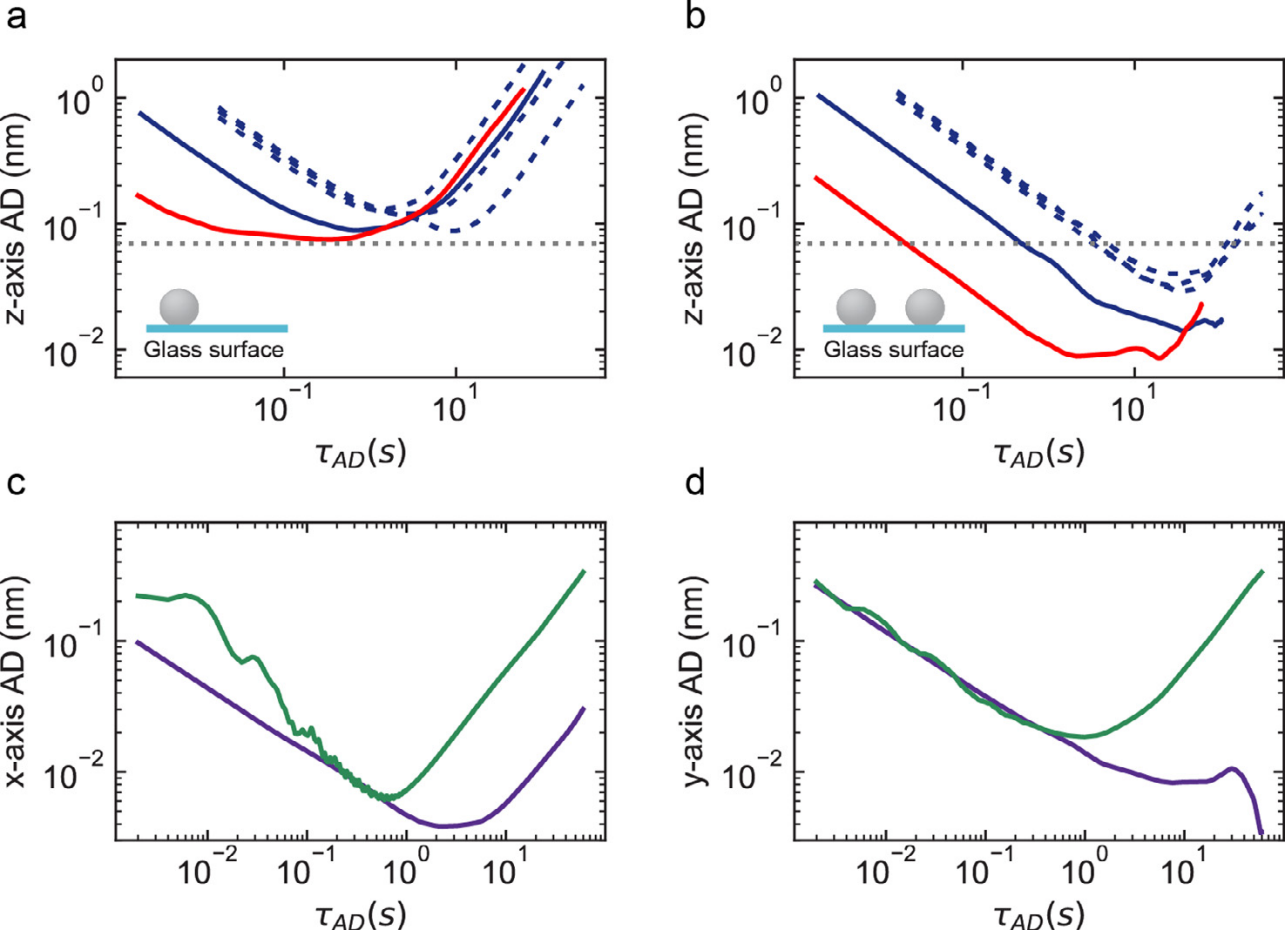
Drift-correction enables sub-Angstrom resolution along the three dimensions for surface-attached beads by removing high frequency noise. (a) AD along the z-axis for a surface-melted 3 µm polystyrene bead acquired using a 50x (blue, 120 nm pixel size) and 100x (red, 60 nm pixel size) magnification, respectively, and at 58 Hz (blue dashed lines) and 500 Hz (blue and red solid lines) acquisition frequency. (b) AD along the z-axis for drift-corrected reference beads. Color code as in (a). The gray horizontal dashed line in (a, b) indicates AD=0.07 nm. **(**c and d) AD along the x- and y-axis for a drift corrected (purple) and non-corrected (green) surface-melted polystyrene bead of 3 µm diameter, imaged using 100x magnification at 500 Hz acquisition frequency. (For interpretation of the references to colour in this figure legend, the reader is referred to the web version of this article.)

**Fig. 4:**
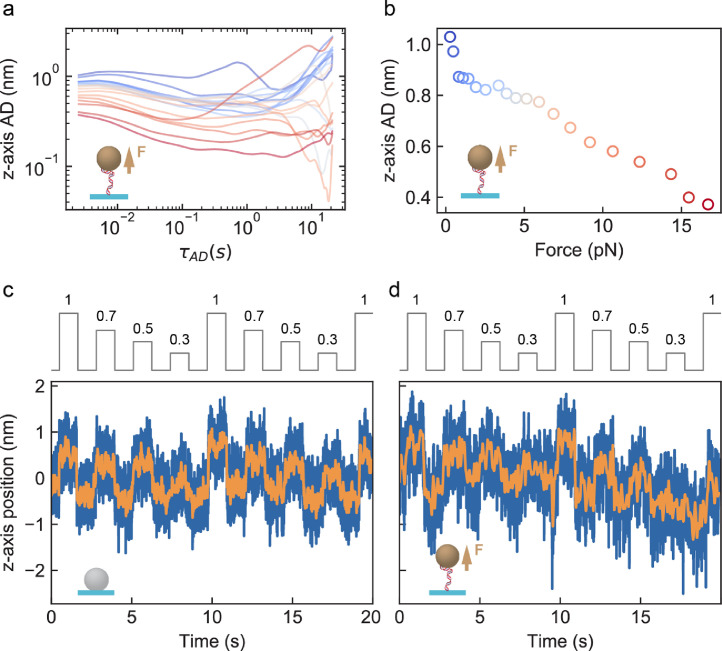
MT resolve sub-nanometer steps using a magnetic bead tethered by a 300 bp dsDNA. (a) z-position AD of a magnetic bead tethered by a 300 bp dsDNA at forces ranging from 0.5 pN (dark blue) to 18 pN (dark red) (400 Hz acquisition frequency). (b) z-axis AD at first frame for τ_AD_ = 2.5 ms as a function of force for the experiment described in (a). (c and d) 1, 0.7, 0.5 and 0.3 nm steps every second (gray line) using (c) a 3 µm polystyrene bead melted on the surface and (d) a magnetic bead tethered to a 300 bp dsDNA under 27 pN force. Raw data (blue) was acquired at 900 Hz for the surface-melted bead and 360 Hz for the tethered magnetic bead and low pass filtered to 25 and 8 Hz (orange), respectively. 100x magnification is used here. (For interpretation of the references to colour in this figure legend, the reader is referred to the web version of this article.)

Fig. 3(a) shows that acquiring with either 50x or 100x magnification, at either 500 Hz or 58 Hz acquisition frequency, the AD along the z-axis plateaus at ∼0.07 nm in absence of reference bead subtraction, indicating a common limiting factor independent of the acquisition frequency and magnification. Fig. 3(b) shows that subtraction of a reference bead position further decrease ADs minimum below 0.07 nm, down to ∼0.01 nm at 500 Hz acquisition frequency and 100x magnification. Interestingly, high frequency noise appears along the x-axis and y-axis, which is also corrected by reference bead subtraction (Fig. 3(c) and (d), respectively). This high frequency noise likely originates from the piezo stage (P-726, Physik Instrumente) on which is mounted the microscope objective, as characterized for optical tweezers [7] and observed in magnetic tweezers using a similar piezo stage [4]. A tethered magnetic bead experiences thermal noise, from the Brownian motion of the surrounding water molecules, which adds up to the position tracking noise [3], [4], [5]. The data presented in Fig. 4(a) shows the AD of a magnetic bead tethered to the glass coverslip surface by a 300 bp DNA molecule, which experiences a stretching force varying from 0.5 pN to 18 pN. Because of the thermal noise contribution, these ADs present a larger amplitude than the ADs for surface attached reference beads (Fig. 3). The AD for a tethered magnetic bead at $\tau_{AD}=2.5\;\text{ms}\;$(1 frame) presented in Fig. 4(b) decreases with the increase in force. We used the high-resolution piezo stage to apply a decreasing step size ramp from 1 nm to 0.3 nm, for a surface-attached reference bead (Fig. 4(c)) and a 300 bp DNA tethered magnetic bead (Fig. 4(d)). All the steps are clearly visible even using the unfiltered trace of the reference bead (Fig. 4(c)), while the 0.3 nm steps are only visible for the filtered trace of the tethered magnetic bead (Fig. 4(d)), opening the way for single DNA base motion observation of molecular motors using magnetic tweezers.

In Supplementary Table 1 we present a list of raw data files, their acquisition conditions, the scripts used for their analysis and the figures generated using the datasets.

## 2 Experimental design, materials, and methods

We tracked the position of: (1) a surface-attached polystyrene reference bead at 50x and 100x magnifications and several acquisition frequencies, subjected to various drift in order to characterize the limiting factors for the best achievable resolution in our assay (Figs. 2 and 3); (2) a magnetic bead tethered by dsDNA under constant force in order to quantify the thermal noise to which the bead is subjected and the relationship between thermal noise and applied force)(Fig. 4(a) and (b)); (4) both immobilized and tethered bead while performing steps with the piezo stage along the z-axis (of 1, 0.7, 0.5, 0.3 nm)(Fig. 4(c) and (d)).

## 3 Magnetic tweezers experimental configuration and force calibration

The magnetic tweezers apparatus is implemented on a custom-built inverted microscope that has already been described elsewhere [1–3] (Fig. 1(a)). The collimated light emitted by an LED (660 nm, 400 mW, LH CP7P, Hechigen, Germany; spherical condenser, NA = 0.79, Thorlabs, Germany) illuminates the sample through the gap between the magnets pair [8]. The applied magnetic field is generated by a pair of vertically aligned permanent magnets (neodymium 5 mm cubes, W-05-G, SuperMagnete, Switzerland) [9] separated by a gap of 1 mm, from which the vertical distance to the sample and the rotation are controlled by two linear motors (Fig. 1(a)), respectively M-126.PD1 and CD-150, which are separately controlled by two USB Mercury controllers (Physik Instrumente, Germany). The field of view is imaged by either a 50x (CFI Plan Achro 50 XH, NA 0.9, Nikon, Germany) or a 100x (CFI Plan Achro 100 XH, NA 1.25, Nikon, Germany) oil immersion objective that is mounted onto a P-726 PIFOC piezo stage controlled by the E-753 piezo controller (Physik Instrumente, Germany). The image is formed by an achromatic doublet (G322304000, f = 200 mm, Qioptiq, Germany) onto a CMOS camera (Dalsa Falcon2 FA-80-12M1H, Stemmer Imaging, Germany), and the image acquisition is controlled using the PCIe 1433 frame grabber (National Instrument, USA). The imaging path from the objective to the camera is tubed using the Thorlabs optical tube and cage system. The CMOS camera has a field of view of 4096 × 3072 pixels and 6 µm pixel size. The magnetic tweezers apparatus interface and the environment of the CPU or GPU-based tridimensional bead position tracking algorithm are written in a custom software (LabView 2016, National Instruments, USA) [1]. For GPU-based tracking, we used a GeForce GTX 1080 (NVIDIA) graphic card.

## 4 DNA construct

The 306 bp dsDNA construct has two digoxigenins at one end and one biotin on the other end, which were inserted by PCR of the λ phage DNA using the primers obtained from biomers.net: 5′-DIG-gtgatattccgtcgc(5′-DIG-)tgctg, 5′-BIO-cacgctcaatctgacaggtg, and the Phusion High-Fidelity DNA Polymerase, following the manufacturers’ instructions (F-530XL, Thermofisher, Germany). The amplified fragment was purified using the MonarchTM PCR Purification Kit (T1030L, New England Biolabs, USA).

## 5 Flow cell assembly

Flow cells were assembled from two glass coverslips (#1, 24 × 60 mm, Menzel GmbH, Germany) separated by a double-layer of Parafilm (P7793, Sigma Aldrich, Germany) carved with a scalpel to form a channel. Inlet and outlet holes were drilled on the top coverslip with a sandblaster (Problast 2, Vaniman, CA, USA). Both coverslips were washed by sonication in a 2% (V/V) Hellmanex III (Z805939-1EA, Sigma Aldrich, Germany) aqueous solution for 15 min, thoroughly rinsed with deionized water and dried at ∼80 °C. Preceding flow cell assembly, the top surface of the bottom coverslip was spread with 4 µl of 3 µm polystyrene bead solution, made from a 1:150 dilution of the stock (LB30, Sigma Aldrich, Germany) in deionized water further diluted 10-times in ethanol. The coverslip was heated at ∼150 °C for 3 min on a hot plate to ensure fixation of the polystyrene beads to served as reference beads during MT experiments. To finally assemble the flow cell, the carved Parafilm was sandwiched between the two coverslips, such that the two holes of the top coverslips were aligned with the channel, and melted at ∼90 °C for ∼30 s.

## 6 Measurement preparation

After mounting the flow cell on the microscope, 3 mL of PBS buffer were flushed through the flow cell. The flow cell was left to equilibrate for a few hours after which measurements on melted reference beads were performed.

For measurements using tethered magnetic beads, anti-digoxigenin (11,333,089,001, Sigma Aldrich, Germany) at 25 µg/mL in PBS buffer was first incubated in the flow channel for 15 min. The flow cell was rinsed the 1 mL PBS, followed by a 5 min incubation deionized water and then in PBS buffer complemented with 600 mM NaCl, and rinsed again with 1 mL PBS buffer. Next, BSA (B9000S, New England Biolabs, USA) at 1 mg/mL in PBS was incubated for 30 min. During BSA incubation, 20 µL of M270 streptavidin-coated superparamagnetic Dynabeads (65,306, Thermofisher, Germany) were washed twice in the rinsing buffer (PBS, 0.1 mg/mL BSA), mixed with 20 µL of DNA at ∼15 pM in rinsing buffer and incubated for a few minutes. The nucleic acids bound to the magnetic beads were flushed in the flow channel and incubated for 5–10 min to ensure attachment of the DNA construct to anti-digoxigenin. Finally, the excess of magnetic beads was removed by flushing copious amounts of rinsing buffer. Non-specifically and weakly attached magnetic beads were detached from the flow channel bottom surface by applying a force of 15 pN while rotating the magnets back and forth and gently tapping the outlet tube and removed by flushing using rinsing buffer.

## 7 Data acquisition and analysis

All relevant acquisition parameters are specified in the Supplementary Table 1. They include: acquisition speed, objective magnification, shutter time, applied force, number of beads, number of beads suitable for analysis, type of beads (tethered or melted) and DNA construct.

The data were analyzed using Python 3.6. Overlapping Allan deviation was calculated using the AllanTools 2019.9 package.

## Appendix Supplementary material

Image, application 1
