# Supplementary material for: High spatiotemporal resolution data from a custom magnetic tweezers instrument
Source: Data Brief. 2020 Mar 12;30:105397. doi: 10.1016/j.dib.2020.105397 (PMC7103768; doi:10.1016/j.dib.2020.105397)
Supplement: Supplementary file 1 [file mmc1.docx]

| File name | Information | Magnifi-cation | Acquisition  frequency (Hz) | Shutter time (ms) | Illumination  (grey levels) | Type of beads | Tether | Force (pN) | Number of beads | Beads # used for figures* | Columns # used for figures** | Figure |
| --- | --- | --- | --- | --- | --- | --- | --- | --- | --- | --- | --- | --- |
| 20191107_33meltedbeads_  58Hz_drift16p65 | x,y,z position | 50x | 58 | 1.1 | 160 | Polystyrene | No | No | 33 | 3 | 11 | 2 a, c, e |
|  |  |  |  |  |  |  |  |  |  | 3, 7 | 11, 23 | 2 b, d, e, f |
| 20191107_33meltedbeads_  58Hz_drift6p65 | x,y,z position | 50x | 58 | 1.1 | 160 | Polystyrene | No | No | 33 | 3 | 11 | 2 a, c, e |
|  |  |  |  |  |  |  |  |  |  | 3, 7 | 11, 23 | 2 b, d, e |
| 20191107_33meltedbeads_  58Hz_drift6p65 | x,y,z position | 50x | 58 | 1.1 | 160 | Polystyrene | No | No | 33 | 3 | 11 | 2 a, c, e |
|  |  |  |  |  |  |  |  |  |  | 3, 7 | 11, 23 | 2 b, d, e |
|  |  |  |  |  |  |  |  |  |  | 4, 7 | 14,23 | 3 a, b |
| 20191107_33meltedbeads_  58Hz_drift1p76 | x,y,z position | 50x | 58 | 1.1 | 160 | Polystyrene | No | No | 33 | 3 | 11 | 2 a, c, e |
|  |  |  |  |  |  |  |  |  |  | 3, 7 | 11, 23 | 2 b, d, e |
| 20191107_33meltedbeads_  58Hz_drift1p4 | x,y,z position | 50x | 58 | 1.1 | 160 | Polystyrene | No | No | 33 | 3 | 11 | 2 a, c, e |
|  |  |  |  |  |  |  |  |  |  | 3, 7 | 11, 23 | 2 b, d, e |
| 20191107_33meltedbeads_  58Hz_drift1p04 | x,y,z position | 50x | 58 | 1.1 | 160 | Polystyrene | No | No | 33 | 3 | 11 | 2 a, c, e |
|  |  |  |  |  |  |  |  |  |  | 3, 7 | 11, 23 | 2 b, d, e |
| 20191107_33meltedbeads_  58Hz_drift0p84 | x,y,z position | 50x | 58 | 1.1 | 160 | Polystyrene | No | No | 33 | 3 | 11 | 2 a, c, e |
|  |  |  |  |  |  |  |  |  |  | 3, 7 | 11, 23 | 2 b, d, e |
|  |  |  |  |  |  |  |  |  |  | 4, 7 | 14, 23 | 3 a, b |
| 20191107_33meltedbeads_  58Hz_drift0p36 | x,y,z position | 50x | 58 | 1.1 | 160 | Polystyrene | No | No | 33 | 3 | 11 | 2 a, c, e |
|  |  |  |  |  |  |  |  |  |  | 3, 7 | 11, 23 | 2 b, d, e |
|  |  |  |  |  |  |  |  |  |  | 4, 7 | 14, 23 | 3 a, b |
| bead_list | x, y coordinates of each bead | - | - | - | - | - | - | - | 33 | All except 9, 10 | 4, 7, 10 …98, 101 except 29, 32 | 2 f |
| 20191107_7meltedbeads_  464Hz_50x | x,y,z position | 50x | 464 | 1.1 | 160 | Polystyrene | No | No | 7 | 1 | 4 | 3 a |
|  |  |  |  |  |  |  |  |  |  | 1, 2 | 4, 7 | 3 b |
| 20191107_2meltedbeads_  500Hz_100x | x,y,z position | 100x | 500 | 1.1 | 155 | Polystyrene | No | No | 2 | 1 | 4 | 3 a |
|  |  |  |  |  |  |  |  |  |  | 1, 2 | 2, 3, 4, 5, 6, 7 | 3 b, c, d |
| 20191114_1tether_  difforce_400Hz | x,y,z position | 100x | 400 | 2.5 | 160 | Magnetic | 300 bp dsDNA | From 0.3 to 18 | 1 | 1 | 4 | 4 a, b |
| 20191114_1tether_difforce_  400Hz_sections | section file |  |  |  |  |  |  |  |  |  |  |  |
| 20191114_1tether_difforce_  400Hz_script | script |  |  |  |  |  |  |  |  |  |  |  |
| 20191028_1meltedbead_  steps_900Hz | x,y,z position | 100x | 900 | 1.1 | 155 | Polystyrene | No | No | 1 | 1 | 4 | 4 c |
| 20191028_1meltedbead_  steps_900Hz_sections | section file |  |  |  |  |  |  |  |  |  |  |  |
| 20191028_1meltedbead_  steps_900Hz_script | script |  |  |  |  |  |  |  |  |  |  |  |
| 20191114_1tether_27pN_  steps_360Hz | x,y,z position | 100x | 360 | 2.75 | 170 | Magnetic | 300 bp dsDNA | 27 | 1 | 1 | 4 | 4 d |
| 20191114_1tether_27pN_  steps_360Hz_sections | section file |  |  |  |  |  |  |  |  |  |  |  |
| 20191114_1tether_27pN_  steps_360Hz_script | script |  |  |  |  |  |  |  |  |  |  |  |

**Supplementary Table 1: Overview of the experimental conditions in which data were acquired.**

* Bead #: bead number

** Column #: column number
